# Non-randomness of the anatomical distribution of tumors

**DOI:** 10.1186/s41236-017-0006-7

**Published:** 2017-12-19

**Authors:** Clare Yu, James Kameron Mitchell

**Affiliations:** 1 Department of Physics and Astronomy, University of California, Irvine, CA 92697-4575 USA; 2 Present address: Department of Physics, Harvard University, Cambridge, MA 02138 USA

**Keywords:** Breast cancer, Anatomical tumor distribution, Vasculature, Upper outer quadrant

## Abstract

**Background:**

Why does a tumor start where it does within an organ? Location is traditionally viewed as a random event, yet the statistics of the location of tumors argues against this being a random occurrence. There are numerous examples including that of breast cancer. More than half of invasive breast cancer tumors start in the upper outer quadrant of the breast near the armpit, even though it is estimated that only 35 to 40% of breast tissue is in this quadrant. This suggests that there is an unknown microenvironmental factor that significantly increases the risk of cancer in a spatial manner and that is not solely due to genes or toxins. We hypothesize that tumors are more prone to form in healthy tissue at microvascular ‘hot spots’ where there is a high local concentration of microvessels providing an increased blood flow that ensures an ample supply of oxygen, nutrients, and receptors for growth factors that promote the generation of new blood vessels.

**Results:**

To show the plausibility of our hypothesis, we calculated the fractional probability that there is at least one microvascular hot spot in each region of the breast assuming a Poisson distribution of microvessels in two-dimensional cross sections of breast tissue. We modulated the microvessel density in various regions of the breast according to the total hemoglobin concentration measured by near infrared diffuse optical spectroscopy in different regions of the breast. Defining a hot spot to be a circle of radius 200 μm with at least 5 microvessels, and using a previously measured mean microvessel density of 1 microvessel/mm^2^, we find good agreement of the fractional probability of at least one hot spot in different regions of the breast with the observed invasive tumor occurrence. However, there is no reason to believe that the microvascular distribution obeys a Poisson distribution.

**Conclusions:**

The spatial location of a tumor in an organ is not entirely random, indicating an unknown risk factor. Much work needs to be done to understand why a tumor occurs where it does.

**Electronic supplementary material:**

The online version of this article (10.1186/s41236-017-0006-7) contains supplementary material, which is available to authorized users.

## Background

### The question of tumor location: Why does a tumor occur where it does?

While the location of a tumor in an organ is often viewed as random, the statistics of the anatomical distribution of tumors indicates that tumor location is not random in the sense that the probability that a tumor will occur in a given region is not proportional to the volume of that region of the organ. For example, more than half of invasive breast tumors occur in the upper outer quadrant of the breast near the armpit (Morris & Kwong, 2004) (see below). Lung tumors occur more than twice as often in the upper lobe compared to the lower lobe of the lung even though the upper and lower lobes have roughly the same volume (Byers et al., 1984). Examination of tissue from prophylactic oophorectomies from women at high risk for ovarian cancer finds that microscopic cancer most commonly occurs in the fimbrae (Crum et al., 2007), i.e., the distal portion of the fallopian tubes near the ovaries, even though the fallopian tubes are histologically the same along their entire length. If we divide the colon at the splenic flexure into a proximal and a distal section (and exclude the rectum), colon cancer tends to be found in the proximal, rather than in the distal, part of the colon even though these two sections are roughly the same length. SEER (Surveillance, Epidemiology, and End Results) data (Siegel et al., 2014) from 2006 to 2010 indicates that 42% of the cases of colon cancer occurred in the proximal colon compared to 23% in the distal colon. The rest (28%) occurred in the rectum. It should be noted that the proximal and distal colon differ in terms of development (embryology), physiology, and molecular biology (Gervaz et al., 2004) but it is not clear whether any of these differences can account for the preference of tumors to occur in the proximal colon.

So why does a tumor occur where it does? The lack of randomness in the anatomical distribution of tumors in various organs suggests that there is an unknown microenvironmental factor that significantly increases the risk of cancer in a spatial manner. Though it has largely been ignored, this question is important since it implies that it is not simply genes or toxins that promote tumor initiation preferentially in some regions of a given organ.

In this paper, we propose that the microvessel density could be a predisposing factor. Our hypothesis is that tumors are prone to develop where there is a high concentration of blood flow that can supply ample oxygen and nutrients to the tumor. We will refer to such regions with a high concentration of microscopic blood vessels as ‘microvascular hot spots’. We will use the breast as an example to discuss our proposition that local variations in the microvascular density could account for the increased tumor incidence in the upper outer quadrant. (A review of this paper with more introductory material is contained in a review article that will appear in *Reports on Progress in Physics*. The review article is by the same authors as this paper and is entitled “The Physical Location of Incipient Cancer: Why Does a Tumor Start Where It Does?”)

### Breast tumors occur most frequently in the upper outer quadrant

**Fig. 1:**
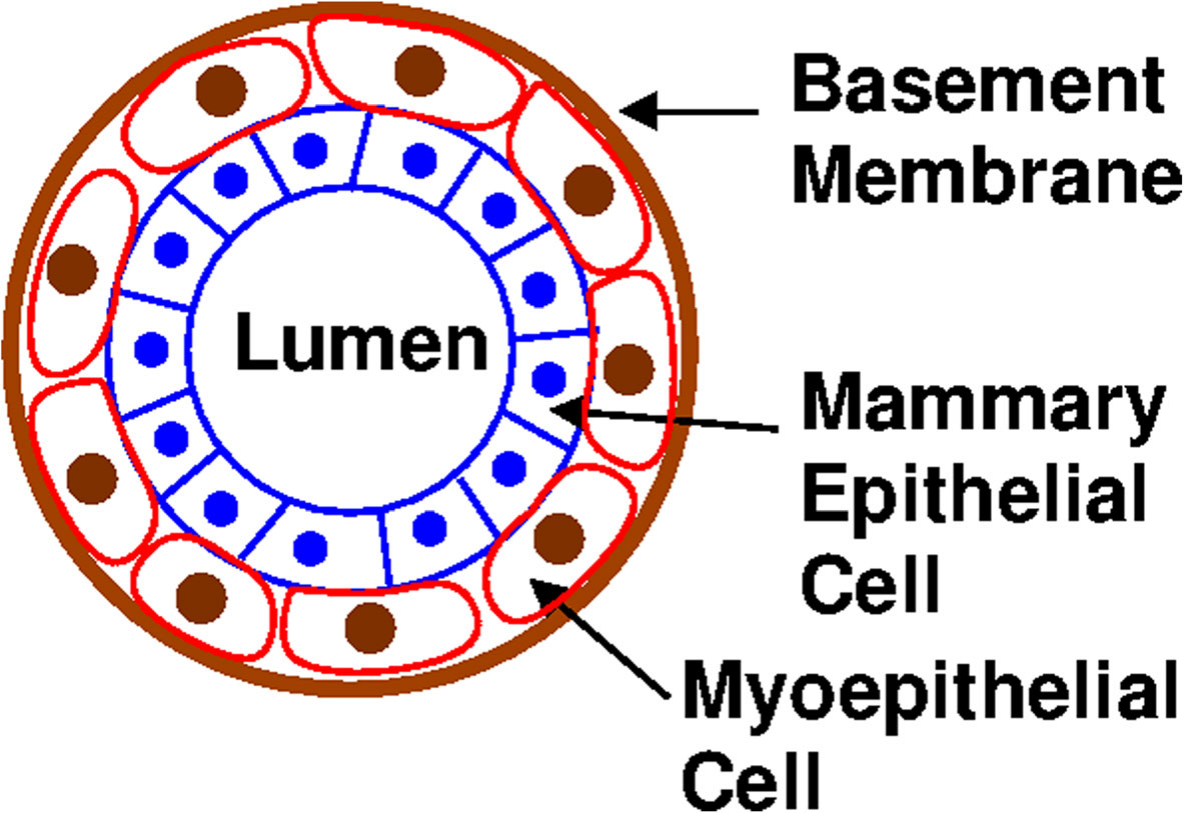
Cross section of a normal milk duct showing the lumen, mammary epithelial cells, myoepitheial cells, and basement membrane

Since the rest of this article will focus on the occurrence of breast tumors in the upper outer quadrant, we will begin with a brief introduction to the breast (Schnitt & Collins, 2018; Love et al., 2015) and breast cancer. The function of the mammary gland is to produce milk. Milk is produced in small grapelike sacs called acini. A cluster of acini is called a lobule and a cluster of lobules is called a lobe. The milk produced in acini flows into a small channel called a ductule. Ductules merge to form milk ducts. Each lobe has one milk duct. If you look at the cross-section of a milk duct (see Fig. 1), it is lined with mammary epithelial cells. Surrounding the mammary epithelial cells is a layer of myoepithelial cells that help to squeeze the milk down the duct. Outside the myoepithelial cells is a tough outer sheath called the basement membrane. It is an example of extracellular matrix and is composed of proteins such as collagen.

Most breast tumors start in the milk ducts or lobules. Typically the mammary epithelial cells start to proliferate and fill up the luminal space in the duct or acini. When they do this to a substantial degree, this is known as ductal carcinoma in situ. This is stage zero cancer. The phrase “in situ” literally means “in place”. In this case, it means that the cells have not broken through the basement membrane. If the epithelial cells continue to proliferate and break out of the duct and through the basement membrane, then this is called invasive ductal carcinoma. “Invasive” means that the cells have invaded tissue where they do not belong.

Analysis of the data for the location of invasive tumors from approximately 137,000 California female breast cancer patients between 1988 and 1999 reveals that 57% of these tumors occur in the upper outer quadrant (UOQ) of the breast near the armpit (Morris & Kwong, 2004). The three other quadrants and the central/areolar/nipple region each account for 15% or less of the tumors as shown in Table 1. If the location of breast tumors were random, then we would expect the probability of a tumor to occur in a region to be proportional to the volume of that region. However, the UOQ certainly does not contain more than half of the breast tissue.

**Table 1:** Location of invasive breast tumors. Data from 137,000 California female beast cancer patients between 1988 and 1999 (Morris & Kwong, 2004). These include stage 1 and higher stages as well as different types of breast cancer

Upper Outer	Upper Inner	Lower Outer	Lower Inner	Nipple, Areola
57%	15%	10%	8%	11%

**Fig. 2:**
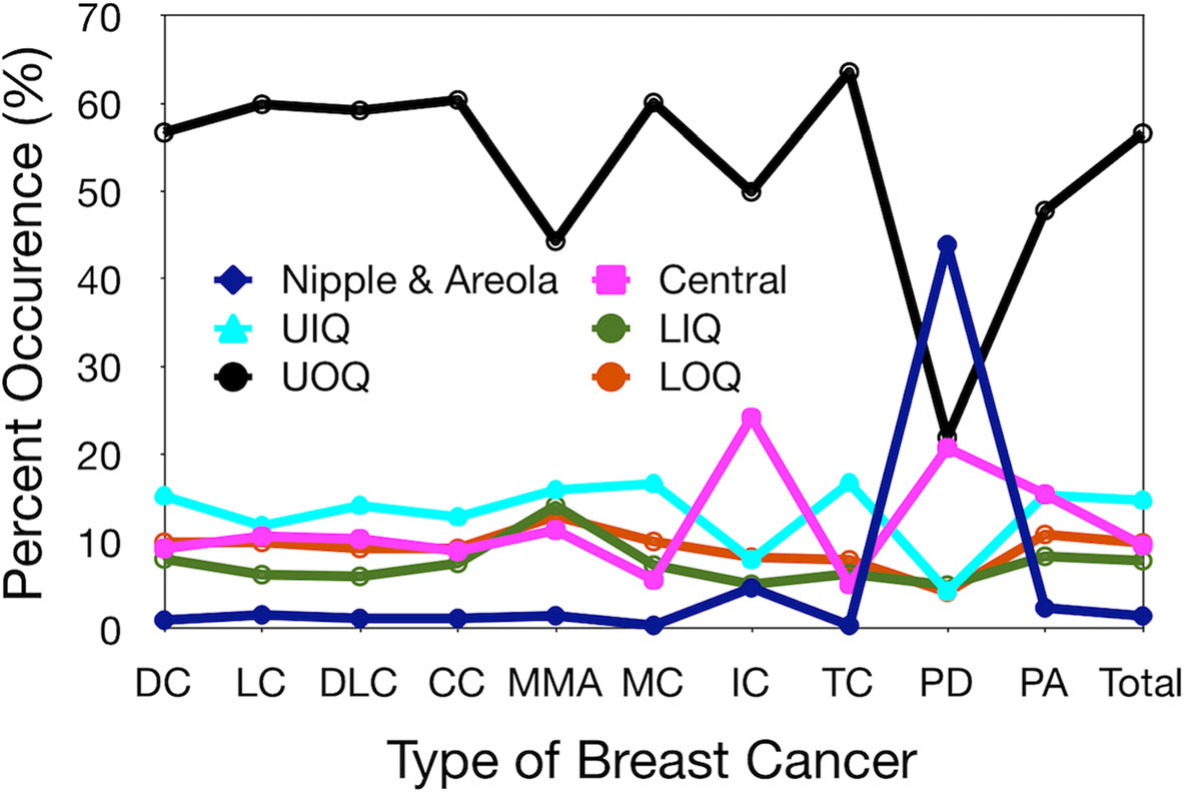
Anatomical site distribution of different types of breast tumors (Morris & Kwong, 2004). Note that almost all types of tumors occur most frequently in the upper outer quadrant (UOQ) with the exception of Paget’s disease (PD) which corresponds to nipple cancer. UIQ = upper inner quadrant, LIQ = lower inner quadrant and LOQ = lower outer quadrant. DC = ductal carcinoma, LC = lobular carcinoma, DLC = ductal lobular carcinoma, CC = comedocarcinoma, MMA = mucinous and mucin-producing adenocarcinoma, MC = medullary carcinoma, IC = inflammatory carcinoma, TC = tubular carcinoma, and PA = papillary adenocarcinoma

If one considers different types of breast cancer, then almost all types of breast tumors occur most frequently in the upper outer quadrant as shown in Fig. 2. The only exception is Paget’s disease which is a type of cancer of the nipple.

## Methods

### Hypothesis: Tumors preferentially form in regions with high microvascular density

Tumors must have blood vessels supplying oxygen and other nutrients if they are to grow beyond a few cubic millimeters. (However, the lack of oxygen, a condition known as hypoxia, can also promote tumor growth. Possible mechanisms for how hypoxia could promote tumor growth include impeding the immune system, selecting for hardier cancer cells that can resist radiation and chemotherapy drugs, and encouraging cancer cells to migrate and invade other tissues (Jain, 2014; Blagosklonny, 2004)). Since oxygen only diffuses 100–150 μm (Fidler et al., 2002; Gray et al., 1953; Tannock, 1968; Brown & Giaccia, 1998; Thomlinson & Gray, 1955), and cytokines like vascular endothelial growth factor (VEGF) diffuse even less due to their size, there must be microvessels within this distance of an incipient tumor. More tissue in the UOQ means more microvessels near which tumors could grow. However, assuming uniform perfusion, the fraction of microvessels in the UOQ should be proportional to the fraction of tissue in that quadrant. So if 35–40% of the tissue is in the upper outer quadrant, then 35–40% of the vasculature (and tumors) should be in the UOQ. However, perfusion is not uniform as indicated by optical measurements (Parbhoo & Seifalian, 1998; Shah et al., 2004; Svensson et al., 2005). Both laser Doppler imaging (Parbhoo & Seifalian, 1998), which measures the skin blood flow, and near infrared diffuse optical spectroscopy (DOS) measurements (Shah et al., 2004; Svensson et al., 2005), which penetrates into the tissue and can measure total hemoglobin concentration in a volume of about 10 cm^3^, find variations in the blood flow between different quadrants of the breast as well as the areolar region. However, prima facie, these variations do not appear to be large enough to account for the preponderance of tumor incidence in the UOQ.

Our hypothesis is that incipient tumors preferentially tend to form at ‘microvascular hot spots’ that correspond to small regions of tissue with a high concentration of microvessels. We hypothesize that perhaps small incipient tumors are more likely to flourish and be vascularized if they are located at “microvascular hot spots” where there is a particularly high density of microvessels. We are referring to hot spots present in disease-free/cancer-free tissue that has not been remodeled by proliferating neoplastic tissue (Carpenter et al., 2011).

We would like to calculate the probability of such hot spots in the different quadrants to see if there is a higher probability for such hot spots in the upper outer quadrant, and to determine if this higher probability is consistent with the higher incidence of tumors in this quadrant. Unfortunately, there are no measurements of the microvascular distribution in the breast, nor have there been measurements of the fraction of breast tissue in each quadrant.

However, as a simple example of such a calculation, we will imagine two-dimensional (2D) cross sections of tissue that have been stained to reveal microvessels as discrete points. Our goal is to calculate the probability *Q* that at least one hot spot occurs in a region of surface area *A*. To illustrate our method of calculation, consider marking a spot on a table, drawing a circle of radius *R* centered at the spot, sprinkling salt on the table and asking what is the probability *P* that at least *n* grains of salt are within the circle. The larger the number of grains required to be in the circle, the smaller the probability of a small circle enclosing that many grains of salt. Suppose we define a hot spot as such a circle with many grains of salt. If we have a big table, divide it into 4 unequal regions and sprinkle the salt evenly on the table, then the probability of at least one hot spot in a region is roughly proportional to the area of the region (as long as NP < < 1 where N is the number of nonoverlapping circles blanketing the region). In order for the probability to not be proportional to the size of the region, the salt must be sprinkled unevenly. The table represents the breast. The four unequal regions represent the four quadrants of the breast. The grains of salt represent the microvessels in a cross section of tissue. The center of the circle represents the tumor location.

**Fig. 3:**
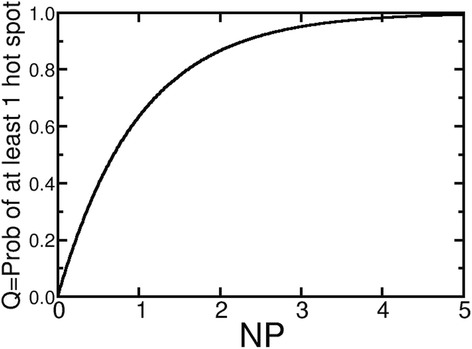
Plot of Q vs the product NP of the number N of trials and the probability P. Note that Q ~ NP for NP < < 1 and Q ~ 1 for NP > > 1. The crossover occurs for NP ~1. Note that the number N of circles filling the region is proportional to the area A of the region. For this plot we used *P* = 1%

Mathematically, suppose we define a hot spot as a circle of radius *R* that contains at least *n* microvessels. Then *N = A*/(*πR* ^2^) is the number of non-overlapping circles (regardless of whether they are a hot spot) that can blanket an area *A*. (Note that *N* may not be an integer.) Let *P* be the probability that a circle is a hot spot. Then (1 – *P*) is the probability that a circle is *not* a hot spot. (1 – *P*)^N^ is the probability that there are no hot spots in *N* circles. *Q* = 1 - (1 – *P*)^N^ is the probability that there is at least one hot spot in *N* circles. Thus, *Q* ≈ *NP* ∝ *AP* if *NP* < < 1. A plot of *Q* versus *NP* is shown in Fig. 3.

As a simple example, suppose a two dimensional cross-section of breast tissue has microvessels distributed according to a Poisson distribution. Then the probability *p*_*m*_(*R,i*) that there are *m* microvessels in a circle of radius *R* in the *i*th region of the breast is given by the Poisson distribution: ${p}_m\left(R,i\right)=\left[{\left\langle m\right\rangle}_i/m!\right]{e}^{\hbox{-} {\left\langle m\right\rangle}_i}$ where 〈*m*〉_*i*_ = *ρ*(*i*)*πR* ^*2*^ is the average number of microvessels in a circle of radius R and *ρ*(*i*) is the areal density of microvessels in the *i*th region of the breast. We divide the breast into five regions: the upper outer quadrant (UOQ), the upper inner quadrant (UIQ), the lower outer quadrant (LOQ), the lower inner quadrant (LIQ), and the areolar region (AR). Then the probability *q*_*n*_(*R*,*i*) that there are at least *n* microvessels in a circle of radius *R* is given by

1 $$ {q}_n\left(R,i\right)=1\hbox{-} \sum \limits_{m= 0}^{n\hbox{-} 1}{p}_m\left(R,i\right) $$

To relate this to the notation above, if we define a hot spot as a circle of radius *R*_*0*_ with at least *n*_*0*_ microvessels, then the probability *P* that a circle is a hot spot is $P={q}_{n_0}\left({R}_0,i\right)$. So the probability *Q*(*i*) that there is at least one hot spot in the *i*th region is given by

2 $$ Q(i)=1\hbox{-} {\left[1\hbox{-} {q}_{n_0}\left({R}_0,i\right)\right]}^{N(i)} $$

where *N*(*i*) = *A*(*i*)/πR_0_^2^ is the number of circles that fit in the area *A*(*i*) of the *i*th region. (Note that *N*(*i*) may not be an integer.) $\sum \limits_{i=1}^5A(i)={A}_T$ where *A*_*T*_ is the total area of a cross section of the breast. Once we obtain *Q*(*i*), we normalize it by the sum over *Q*(*i*) to obtain the fractional probabilities *Q*_*f*_(*i*) that there is at least one hot spot in the *i*th region: ${Q}_f(i)=Q(i)/\sum \limits_{i=1}^5Q(i)$.

We modulated the density of microvessels in each region of the breast using infrared measurements of the total (both oxygenated and deoxygenated) mean hemoglobin concentration in the various breast regions for postmenopausal women (Shah et al., 2004). To calculate the microvessel density modulation factor *f*(*i*) for the *i*th region, we do the following. Let *h*(*i*) be the total mean hemoglobin concentration measured in the *i*th region. We estimated the hemoglobin mass *m*_h_(*i*) in the *i*th region using *m*_h_(*i*) = *h*(*i*) *V*_*b*_ *b*(*i*) where *V*_*b*_ is the volume of the tissue in the entire breast, and *b*(*i*) is the fraction of tissue in the *i*th region of the breast. The total breast volume *V*_*b*_ will cancel out later so its value does not matter. The total hemoglobin mass *M*_*h*_ in the breast is ${M}_T=\sum \limits_i{m}_h(i)$ . The mass fraction *F*_*h*_ of hemoglobin in the *i*th region is *F*_*h*_(*i*) = *m*_*h*_(*i*)/*M*_*T*_. We take the ratio the hemoglobin mass fraction to the breast tissue fraction to get *f*(*i*), the microvessel density modulation factor: *f*(*i*) = *F*_*h*_(*i*)/*b*(*i*). The microvessel density *ρ*(*i*) in the *i*th region is given by *ρ*(*i*) = *f*(*i*)*ρ*_*0*_ where *ρ*_*0*_ is the measured areal density of microvessels. Measurements of the 2D density *ρ*_*0*_ of microvessels in breast tissue have found values of 1 microvessel/mm^2^ (Carpenter et al., 2011) and 61 microvessels/mm^2^ (El-Gohary et al., 2009).

## Results

**Fig. 4:**
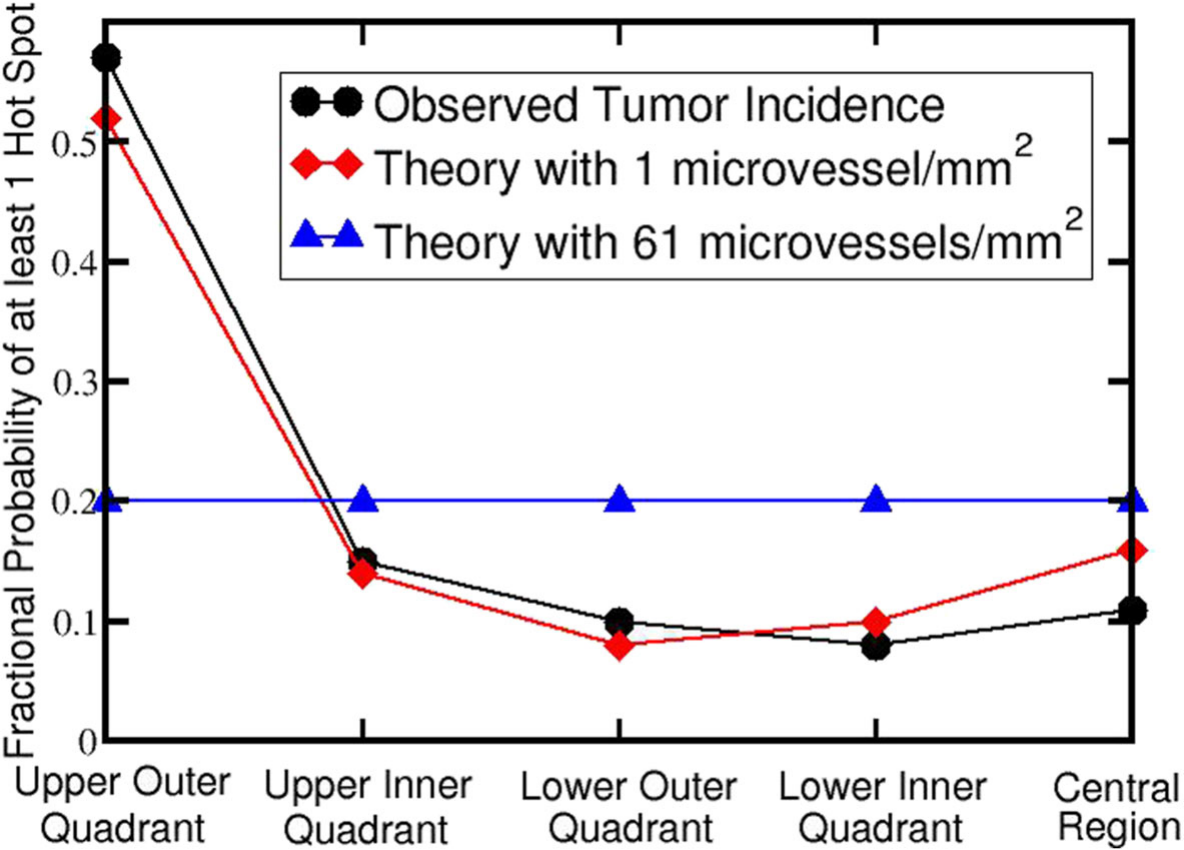
Comparison of the observed distribution of breast tumor locations from (Morris & Kwong, 2004) with the fractional probability of at least one hot spot in the different regions of the breast assuming a Poisson distribution of microvessels in 2D. In our simulation, the sampling circles had a radius of 200 μm. A hot spot was defined as a sampling circle with at least 5 microvessels. The overall cross section of the breast was assumed to be a circle with a radius of 7 cm. The fraction of tissue in the upper outer quadrant (UOQ), upper inner quadrant (UIQ), lower outer quadrant (LOQ), lower inner quadrant (LIQ), and central (nipple) region were assumed to be 0.38, 0.14, 0.24, 0.19, and 0.05, respectively. (Since the fraction of breast tissue in the different regions of the breast has not been measured, these numbers are based on estimates from Dr. Karen Lane, a breast surgeon at the University of California Irvine Medical Center.) To test our model, we used the two published 2D mean microvessel densities of 1 (Carpenter et al., 2011) and 61 (El-Gohary et al., 2009) microvessels/mm^2^. We modulated the microvessel density in various regions of the breast according to the total hemoglobin concentration measured by near infrared diffuse optical spectroscopy in different regions of the left breast of postmenopausal women (Shah et al., 2004), i.e., we used the following values for the total hemoglobin: *h*(UOQ) = 16 μM, *h*(LOQ) = 12 μM, *h*(LIQ) = 13 μM, *h*(UIQ) = 15 μM, and *h*(AR) = 19 μM

Using the procedure described above, we have calculated the fractional probabilities for at least one hot spot in the various regions of the breast assuming a Poisson distribution of microvessels in a 2D cross section of breast tissue. A hot spot was defined as a circle of radius 200 μm with at least 5 microvessels. Our results are shown in Fig. 4 along with the observed tumor incidence (Morris & Kwong, 2004) from Fig. 2.

Notice that there is good agreement of the observed tumor incidence location in the case of 1 microvessel/mm^2^ (Carpenter et al., 2011).

### Parameter dependence

The sensitivity of our analysis to model parameters is implicitly contained in the equations. Human anatomy and physiology as well as experimental measurements restrict many of the parameters to a rather narrow range, e.g., the radius of the breast, the fractional amount of tissue in the quadrants of the breast, the variation in the total hemoglobin in various regions of the breast, etc. We are free to choose the parameters associated with our definition of a sampling circle and a hot spot. In the supplement (Additional file 1), we show that our results are rather insensitive to the radius of a hot spot and to the minimum number of microvessels in a hot spot as long as *n* ≥ 4. For *n* < 4, it is easy to meet the requirements for a microvascular hot spot and there is a high probability that every region of the breast will have at least one hot spot.

## Discussion

### Microvessel density and the oxygen diffusion length

We found good agreement between the observed tumor incidence location and the fractional probability of hot spots for a density of 1 microvessel/mm^2^ (Carpenter et al., 2011). However, this would imply a mean spacing of 1 mm between microvessels, a value that seems slightly high if the oxygen diffusion length is 100 to 200 μm (Fidler et al., 2002; Gray et al., 1953; Tannock, 1968; Brown & Giaccia, 1998; Thomlinson & Gray, 1955). Keep in mind that the oxygen diffusing into a space between 2 microvessels covers about 200–400 μm. A microvessel density of 61 microvessels/mm^2^ (El-Gohary et al., 2009) corresponds to a mean spacing of 128 μm, which seems more consistent with 100 to 200 μm as the oxygen diffusion length (see below), but leads to a high probability of a hot spot occurring in each region with our definition of hot spots.

The values we used for the microvessel density were measured in breast tissue so they are both physiologically realistic. However, although the value of 100 to 200 μm is widely cited (Fidler et al., 2002; Gray et al., 1953; Tannock, 1968; Brown & Giaccia, 1998; Thomlinson & Gray, 1955), we know of no direct measurements of the diffusion length of oxygen in living tissue. The values cited in the literature are inferred from indirect measurements. For example, Fidler et al. (Fidler et al., 2002) note that dividing tumor cells tend to be within 75 μm of a blood vessel while apoptotic cells in a tumor are twice as far (160–170 μm) from a blood vessel. Earlier researchers reported that non-necrotic cells in corded tumors (i.e., tumors in the shape of a solid rod) were never further than 100–200 μm from a blood vessel (Thomlinson & Gray, 1955). However, these indirect measurements assume that oxygen is the limiting factor whereas lack of an essential nutrient like glucose or the accumulation of high concentrations of lactic acid could result in necrosis (Thomlinson & Gray, 1955). In addition, the microvessel density varies from organ to organ due to the differing oxygen demands of different types of cells and tissues. For example, fat does not need as much oxygen as muscle.

### The need for measurements of the microvessel density distribution and fraction of breast tissue in various regions

The simple example above assumed a Poisson distribution, but this is not a crucial assumption. Indeed, there is no reason to believe that the Poisson distribution is the actual microvessel density distribution. Other spatial distributions of the microvasculature could also give the observed tumor incidence. That is why it would be useful to measure the microvessel density distribution in all five regions for both 2D and 3D in normal breast tissue obtained from autopsies and prophylactic mastectomies as well as breast reduction surgery. It is important to use normal breast tissue that has not been remodeled by proliferating neoplastic tissue. For 2D tissue slices, the blood vessels could be stained and then one could count the number of blood vessels in a series of non-overlapping circles to obtain a histogram of the density distribution in units of number of microvessels per unit area, e.g., per square mm. The radii of the circles could be comparable to the oxygen diffusion length.

The 3D microvessel density is defined as the length of microvessels per unit volume. Imagine having a 3D vascular network and filling the space with nonoverlapping sampling spheres. The microvessel density equals the length of microvessels inside a sphere divided by the volume of the sphere. A map of the 3D vascular network in breast tissue has never been conducted because of the prohibitive cost of examining a z-stack of 5-μm thick tissue slices using traditional methods. However, it should be possible to map out the 3D vascular structure using advanced optical methods similar to those that have been used to map out a cubic millimeter of the 3D angioarchitecture of the murine vibrissa cortex (Blinder et al., 2013).

We made estimates of the fraction of tissue in each of the 4 quadrants and in the nipple/areolar/central region because this has not been previously measured. However, measurements can and should be done on disease-free human specimens using tissue from prophylactic mastectomies and autopsies. Images from breast magnetic resonance imaging (MRI) are another way to determine the fraction of tissue in different quadrants. Patients undergoing a breast MRI lie prone with their breasts hanging in a pendulous fashion. However, in MRI images, it is sometimes difficult to know exactly where to delineate the boundary between the different quadrants since patients may be slightly tilted, e.g., because they lean more on one elbow than the other.

### Modulation of microvessel density

We modulated the density of microvessels in each region of the breast using infrared measurements of the total mean hemoglobin concentration in the various breast regions for postmenopausal women (Shah et al., 2004). The total hemoglobin measured using near infrared imaging is in both capillaries and larger blood vessels. (Note that oxygen diffuses out of both capillaries as well as pre-capillary vessels (arterioles) (Pittman, 2011). This is why we refer to microvessels rather than capillaries.) In our calculation we do not need to assume that the total hemoglobin is contained entirely in the microvessels. Rather we just assumed that the region-to-region variation of the microvessel density is mirrored in the spatial modulation of the total hemoglobin.

### Previous hypotheses for increased breast tumor incidence in UOQ are problematic

There have been a number of other hypotheses to explain why the preponderance of breast tumors are found in the upper outer quadrant. These include the following:

[A] *Distribution of breast tissue mass:* The conventional wisdom is that there are more tumors in the upper outer quadrant because there is more tissue in that quadrant since the breast is shaped like a teardrop with axillary tissue under the arm in the vicinity of the armpit (Haagensen, 1986; Lee, 2005). However, our analysis indicates that if the 57% tumor incidence were random, this would require 64% of the breast tissue to be in the upper outer quadrant, while certainly less than half of the breast tissue is in the upper outer quadrant. Furthermore, it is not clear that bigger breasts have a higher risk of breast cancer than smaller breasts (Jansen et al., 2014). Several studies find no evidence that increased breast size is associated with increased risk of breast cancer (Wynder et al., 1960; Katariya et al., 1974; Kolonel et al., 1986; Senie et al., 1993; Koch et al., 2004), while others found indications of a positive correlation (Scutt et al., 1997; Hsieh & Trichopoulos, 1991; Joensuu et al., 1992; Kato et al., 1995). Still other studies found that women with smaller breasts were at increased risk (Tavani et al., 1996; Thurfjell et al., 1996). It is worth noting that compared to small breasts, large breasts tend to have more fat but a comparable amount of fibroglandular tissue. (Fibroglandular tissue is where tumors tend to start.) This is consistent with the fact that women with large breasts and small breasts tend to produce the same amount of milk (Edgar, 2005).

[B] *Food additives and environmental toxins*: A higher incidence in the upper outer quadrant has also been documented in third world countries, e.g., the upper outer quadrant incidence is ~51% in India (Dinshaw et al., 2005), ~40% among West Indian women in Trinidad (Raju & Naraynsingh, 1989), ~54% in Goiania in Brazil (Nunes et al., 2011), ~30–48% in Nigeria (Adesunkanmi et al., 2006), and ~50% between 1927 and 1946 in Costa Rica (De Girolami & Luros, 1958). These percentages are all comparable to that found in the large study of California breast cancer patients between 1988 and 1999 (Morris & Kwong, 2004). This implies that modern food additives and environmental toxins cannot explain the higher tumor incidence in the upper outer quadrant. Interestingly, the tumor incidence in the UOQ in England and Wales has slowly been rising from 48% in 1979 to 53% in 2000 (Darbre, 2005).

[C] *Deodorants and antiperspirants*: It has been suggested that the aluminum salts in underarm antiperspirants and deodorants can mimic estrogens and increase the risk of cancer (Darbre & Charles, 2010), but there is no real evidence to support this and it has largely been discounted (Namer et al., 2008). In addition, the higher tumor incidence in the UOQ in third world countries (Dinshaw et al., 2005; Raju & Naraynsingh, 1989; Nunes et al., 2011; Adesunkanmi et al., 2006; De Girolami & Luros, 1958) and between 1927 and 1946 in Costa Rica (De Girolami & Luros, 1958) implies that deodorants and antiperspirants are not the cause. Furthermore, in mammals other than humans, spontaneous mammary tumors are most likely to occur near the legs. (Spontaneous mammary tumors refer to those not caused artificially, e.g., by introducing by a virus.) In dogs, 40–60% of mammary tumors occur in the pair of glands located most caudally, i.e., near the hind legs (Bostock, 1975; Cotchin, 1958; Fidler & Brodey, 1967; Cotchin, 1957; Huggins & Moulder, 1944). In cats (Cotchin, 1957; Hayden & Nielsen, 1971) and mice (Prehn et al., 1954; Pullinger, 1952), most spontaneous mammary tumors occur in the anterior mammary glands, i.e., those closest to the front legs. Most tumors in female rats are benign mammary tumors, and the most common locations of these tumors are in the armpits and in the belly or groin area (Ducummon, 1995). Since the use of antiperspirants and deodorants cannot explain the higher incidence of spontaneous tumors in the mammary glands near the limbs of mammals (Bostock, 1975; Cotchin, 1958; Fidler & Brodey, 1967; Cotchin, 1957; Huggins & Moulder, 1944; Hayden & Nielsen, 1971; Prehn et al., 1954; Pullinger, 1952), this is unlikely to be the explanation.

[D] *Mechanical motion*: The upper outer quadrant is closer to the arms and shoulders. Furthermore, as we mentioned above, spontaneous tumors in other mammals tend to occur near the limbs (Bostock, 1975; Cotchin, 1958; Fidler & Brodey, 1967; Cotchin, 1957; Huggins & Moulder, 1944; Hayden & Nielsen, 1971; Prehn et al., 1954; Pullinger, 1952). So one might wonder whether arm motion could be linked to breast cancer. However, we do not believe that arm motion is a risk factor because, to the best of our knowledge, there does not appear to be any dorsal-ventral asymmetry in the occurrence of breast tumors, i.e., there does not seem to be any increased tumor incidence near (or far from) the chest wall muscles.

[E] *Temperature*: One might argue that the upper outer quadrant is near the armpit which is warmer other parts of the body. However, temperature does not appear to be a factor. Infrared imaging measurements indicate that the upper outer quadrant is neither hotter nor colder than the three other quadrants (Head et al., 2001).

[F] *Lymph*: The lymph system can be thought of as a sewage system for the cells, though it is also a crucial component of the immune system. Cells dump waste products into the surrounding interstitial fluid which is mostly taken up by the venous system with a few percent taken up by the lymph microvessels as lymph fluid. One might be concerned that the toxins and waste products in the lymph could be carcinogenic. In most women, all the areas of the breast drain through the lymph vessels in the upper outer quadrant to the axillary lymph nodes under the arm, and to a lesser extent to the lymph nodes in the internal mammary chain along the sternum (Estourgie et al., 2004; Suami et al., 2008). Lymph drainage through the upper outer quadrant may mean that there is a higher concentration of waste products in this quadrant, but it is not clear how this drainage could adversely affect mammary epithelial cells and other cells outside of the lymph vessels. (As an analogy, the sewage system in your home does not affect your health as long as the sewage stays in the sewage pipes.) Furthermore, ductal carcinoma in situ (DCIS) and lobular carcinoma in situ (LCIS) only involve the lymph system in 1–2% of cases (Wax, 2012), and yet both DCIS and LCIS occur more often in the upper outer quadrant than anywhere else (Morris & Kwong, 2004). (Recall that DCIS and LCIS are confined within the milk ducts and lobules, and have not broken through the basement membrane. So the abnormal cells associated with DCIS and LCIS are not in contact with lymph fluid that is confined in lymph vessels.)

### Implications

If our hypothesis is correct, then this means that the regions with a high density of microvessels are at a greater risk of developing tumors. This would apply not just to breast cancer but to other organs as well. Microvascular hot spots would also increase the risk of being sites of metastasis. This may explain why tumors that metastasize to the breast from an extramammary primary tumor elsewhere in the body preferentially occur in the upper outer quadrant (Toombs & Kalisher, 1977; Lee et al., 2010) with an UOQ incidence of about 66% (Toombs & Kalisher, 1977).

If our hypothesis that tumors are prone to form where there are microvascular hot spots proves true, then there could be important clinical implications. For example, it might help to explain why wounding the cellular microenvironment promotes tumorigenesis (Dolberg & Bissell, 1984). While wounding promotes inflammation and inflammation is known to increase cancer risk (Coussens & Werb, 2002), the remodeling of the vasculature during healing could produce microvascular hot spots that would also promote tumor recurrence. This could help to explain the peak in cancer relapses within 2 to 3 years after resection of tumors in breast (Retsky et al., 2012), prostate (Hanin & Zaider, 2011; Weckermann et al., 2009), lung (Demicheli et al., 2012) and pancreatic (Deylgat et al., 2011) cancers as well as osteosarcoma (Tsunemi et al., 2003) and melanoma (Tseng et al., 2009). If microvascular hot spots increase the risk of recurrence of cancer, then perhaps therapeutics could be found to prevent the formation of microvascular hot spots during wound healing after surgery. In the case of breast cancer a small clinical study has shown that one-time perioperative use of the non-steroidal anti-inflammatory drug (NSAID) ketorolac cuts the risk for early recurrence by half (Retsky et al., 2012; Forget et al., 2010). This could be due to a reduction in the inflammatory response and/or in the regrowth of vasculature without generation of microvascular hot spots.

### Diffuse optical spectroscopic imaging could identify hot spots

Infrared imaging in the form of Diffuse Optical Spectroscopic Imaging (DOSI) could be used to identify microvascular hot spots and hence, patients and regions with a higher risk of cancer. This technique uses diffuse scattering of near-infrared light to assess the local concentrations of metabolites such as oxygenated and deoxygenated hemoglobin with a spatial resolution of approximately 1 cm (Shah et al., 2001; Cerussi et al., 2001; O'Sullivan et al., 2013). Hand-held DOSI scanning devices have been developed to perform whole breast scanning of normal breast tissue (Tromberg et al., 2016). If imaging techniques such as DOSI could identify hot spot regions at high risk for breast cancer, then local treatment could be applied to minimize risk. For example, milk ducts could be ablated in these hot spot regions using breast-conserving prophylactic intraductal therapy. In this therapeutic approach, selected agents are injected into designated ducts, resulting in extensive destruction of mammary epithelial cells (Murata et al., 2006; Stearns et al., 2011).

### Application of hypothesis to tumors in other organs

While we believe that our hypothesis of microvascular hot spots can be applied to tumors in other organs, we are not able to apply our simulation to other types of tumors due to a lack of data on the spatial distribution of blood flow in other organs. The perfusion data (Shah et al., 2004) that we used for the breast was obtained with near infrared diffuse optical spectroscopy (Shah et al., 2001; Cerussi et al., 2001). Infrared radiation can penetrate a small distance below the skin, making the blood flow in the breast is accessible but this is not practical for organs such as the colon, lungs, etc.

## Conclusions

Although tumor location is viewed as random, the statistics of the anatomical location of tumors in organs such as the breasts, lungs, colon and fallopian tubes/ovaries indicate a preference for some anatomical regions over others. The question of why a tumor occurs where it does in an organ cannot be addressed solely in terms of genetics or environmental factors, implying that there is another risk factor for cancer that is not appreciated.

Our hypothesis is that tumors tend to occur at ‘microvascular hot spots’ where there is an unusually high microvessel density in normal tissue. These microvessels would supply ample oxygen and nutrients to a budding tumor, as well as have receptors for factors that promote angiogenesis such as VEGF. In this paper we focused on breast cancer and the observation that about 60% of breast tumors occur in the upper outer quadrant (Morris & Kwong, 2004) even though the upper outer quadrant comprises only 35–40% of the breast tissue.

To demonstrate the plausibility of our hypothesis, we did a calculation to estimate the fractional probability that there is at least one microvascular hot spot in each region of the breast assuming a Poisson distribution of microvessels in 2D cross sections of breast tissue. We modulated the microvessel density in various regions of the breast according to the total hemoglobin concentration measured by near infrared diffuse optical spectroscopy in different regions of the breast of postmenopausal women (Shah et al., 2004). For a mean microvessel density of 1 microvessel/mm^2^ (Carpenter et al., 2011), we find good agreement of the fractional probability of at least one hot spot with the observed invasive tumor occurrence (Morris & Kwong, 2004) in different regions of the breast. Of course, there is no reason to believe that microvessels have a Poisson distribution, and much work needs to be done to measure the microvessel density distribution as well as the fraction of breast tissue in different regions of the breast.

If our hypothesis that tumors are prone to form where there are microvascular hot spots proves true, it might help to explain explain why wounding the cellular microenvironment promotes tumorigenesis (Dolberg & Bissell, 1984) and why cancer recurrence is highest in the first 2 to 3 years after tumor resection (Retsky et al., 2012; Hanin & Zaider, 2011; Weckermann et al., 2009; Demicheli et al., 2012; Deylgat et al., 2011; Tsunemi et al., 2003; Tseng et al., 2009). While inflammation is associated with wound healing and is known to increase cancer risk (Coussens & Werb, 2002), the remodeling of the vasculature during healing could produce microvascular hot spots that could also promote tumor recurrence. If hot spots are a risk factor, then near infrared imaging such as DOSI could be used to identify hot spots and prophylactic measures could be implemented.

In summary, our paper is largely speculative. Our goal has not been to solve a problem, but rather to point out that there is an important problem that has largely been ignored. Namely, that tumor location in an organ is not entirely random, indicating that there are cancer risk factors that have largely been ignored. Our hope is that much more research will be done to understand why tumors occur where they do.

## Additional file

Additional file 1: Parameter Sensitivity. (PDF 282 kb)

## Abbreviations

2D: Two-dimensional
3D: Three-dimensional
CC: Comedocarcinoma
DC: Ductal carcinoma
DCIS: Ductal carcinoma in situ
DLC: Ductal lobular carcinoma
DOS: Diffuse Optical Spectroscopy
DOSI: Diffuse Optical Spectroscopic Imaging
IC: Inflammatory carcinoma
LC: Lobular carcinoma
LCIS: Lobular carcinoma in situ
LIQ: Lower outer quadrant
LOQ: Lower outer quadrant
MC: Medullary carcinoma
MMA: Mucinous and mucin-producing adenocarcinoma
PA: Papillary adenocarcinoma
PD: Paget’s disease
TC: Tubular carcinoma
UIQ: Upper inner quadrant
UOQ: Upper outer quadrant
VEGF: Vascular endothelial growth factor
