# Supplementary material for: Non-randomness of the anatomical distribution of tumors
Source: Cancer Converg. 2017 Dec 19;1(1):4. doi: 10.1186/s41236-017-0006-7 (PMC5876694; doi:10.1186/s41236-017-0006-7)
Supplement: Supplementary file 1 — Parameter Sensitivity. (PDF 282 kb) [file 41236_2017_6_MOESM1_ESM.pdf]

Supplement to
Non-Randomness of the Anatomical Distribution of Tumors

Parameter Sensitivity

In this supplement, we show the sensitivity of our results to the parameters used. Figure S1 shows that the dependence on the radius of the sampling circles is weak.

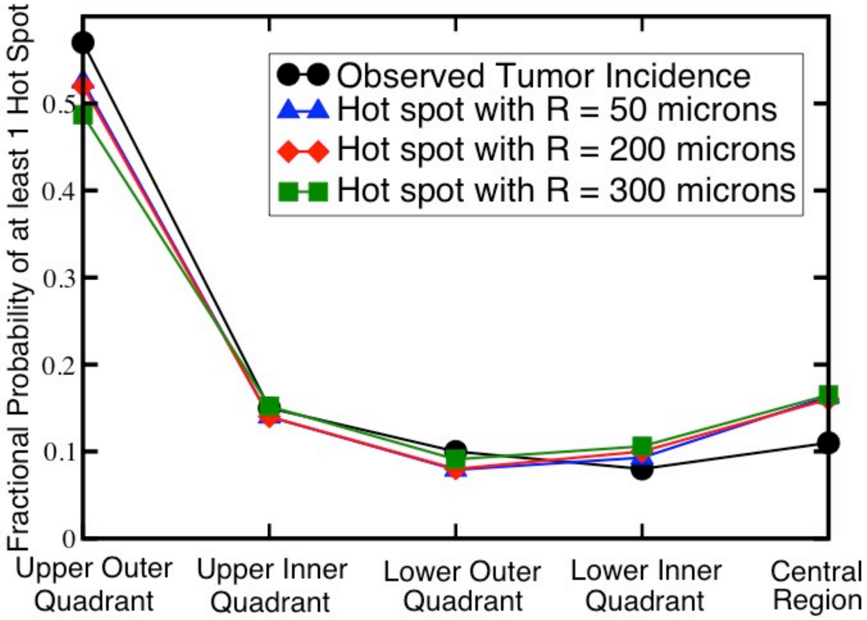

Figure S1: Dependence on the radius of the sampling circle. Comparison of the observed distribution of breast tumor locations from (1) with the fractional probability of at least one hot spot in the different regions of the breast assuming a Poisson distribution of microvessels in 2D. In our simulation, the sampling circles had varying radii as indicated in the legend. A hot spot was defined as a sampling circle with at least 5 microvessels. The 2D mean microvessel density was set to 1 microvessel/mm^2 (2). The rest of the parameters are the same as in Figure 4 in the main text.

The dependence on the definition of the minimum number of microvessels in a microvascular hot spot is shown in Figure S2. A hot spot is defined as a sampling circle with at least *n* microvessels. In Figure S2 the radius of the circle is 200 microns which is comparable to the oxygen diffusion length (3-7). If n ≤ 3 microvessels, then the definition of a hot spot is easily satisfied and there is a good chance that every region of the breast will have at least one hot spot. For *n* ≥ 4, then the fractional probability that there is at least one hot spot in a region of the breast is similar to that of *n* = 5 which matches well with the observed tumor incidence.

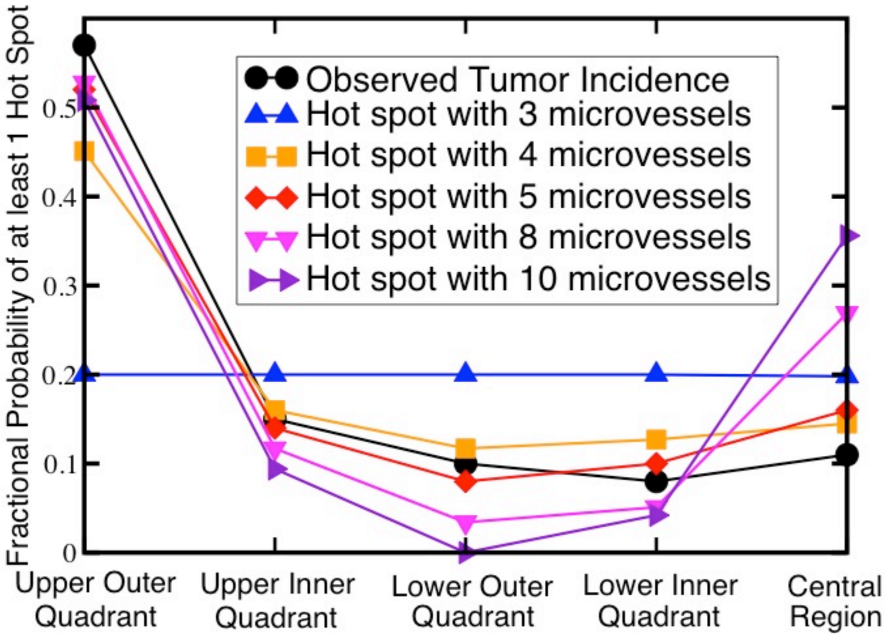

Figure S2: Dependence on minimum number of microvessels in a microvascular hot spot. Comparison of the observed distribution of breast tumor locations from (1) with the fractional probability of at least one hot spot in the different regions of the breast assuming a Poisson distribution of microvessels in 2D. In our simulation, the sampling circles had a radius of 200 microns. A hot spot was defined as a sampling circle with at least n microvessels where the value of n is shown in the legend. The 2D mean microvessel density was set to 1 microvessel/mm^2 (2). The rest of the parameters are the same as in Figure 4 in the main text.

1. Morris CR, Kwong KL. Breast Cancer in California, 2003. Sacramento, CA: California Department of Health Services, Section CS; 2004.
2. Carpenter PM, Chen WP, Mendez A, McLaren CE, Su MY. Angiogenesis in the progression of breast ductal proliferations. International journal of surgical pathology. 2011;19(3):335-41.
3. Fidler IJ, Yano S, Zhang RD, Fujimaki T, Bucana CD. The seed and soil hypothesis: vascularisation and brain metastases. The lancet oncology. 2002;3(1):53-7.
4. Gray LH, Conger AD, Ebert M, Hornsey S, Scott OC. The concentration of oxygen dissolved in tissues at the time of irradiation as a factor in radiotherapy. The British journal of radiology. 1953;26(312):638-48.
5. Tannock IF. The relation between cell proliferation and the vascular system in a transplanted mouse mammary tumour. British journal of cancer. 1968;22(2):258-73.
6. Brown JM, Giaccia AJ. The unique physiology of solid tumors: opportunities (and problems) for cancer therapy. Cancer research. 1998;58(7):1408-16.

7. Thomlinson RH, Gray LH. The histological structure of some human lung cancers and the possible implications for radiotherapy. British journal of cancer. 1955;9(4):539-49.
